# A torted wandering spleen: a case report

**DOI:** 10.1186/1752-1947-8-133

**Published:** 2014-05-01

**Authors:** Aman Sharma, Gisella Salerno

**Affiliations:** 1 FY2 in Paediatric Surgery at John Radcliffe, Headley Way, Oxford OX3 9DU, UK; 2 Wexham Park General Surgery Hospital, Slough, UK

**Keywords:** Gastrosplenic, Hypermobile, Infarcted, Pedicle, Splenectomy, Splenorenal, Torted

## Abstract

**Introduction:**

A torted wandering spleen is a rare clinical occurrence with fewer than 500 cases reported and an incidence of less than 0.2%. It is brought about through laxity of the gastrosplenic and splenorenal ligaments; however, the precise aetiology remains unknown. It can prove to be a diagnostic challenge with high mortality if misdiagnosed.

**Case presentation:**

We present the case of a 27-year-old woman of Arabic ethnicity, who complained of a short history of severe abdominal pain with the background of recurrent abdominal pain and vomiting. An abdominal computerized tomography scan revealed a torted wandering spleen. This required a splenectomy due to splenic infarction.

**Conclusion:**

This report highlights the investigations and management necessary for a patient who presents with an ischaemic torted wandering spleen.

## Introduction

A wandering spleen is a rare clinical occurrence with fewer than 500 cases reported and an incidence of less than 0.2% [1,2]. The spleen is an important component of the reticuloendothelial system, which is involved in immunological defence and can serve as a storage site for red blood cells [3].

The spleen is normally supported by the gastrosplenic, splenorenal and splenocolic ligaments, whereby failure of attachment of these ligaments to the spleen’s overlying peritoneum results in a hypermobile spleen [3,4]. All cases of a wandering spleen have been found associated with a long splenic pedicle which consists of the splenic vessels and the tail of the pancreas [2-4].

A wandering spleen can be either congenital or acquired. In the congenital condition the ligaments fail to develop properly, whereas in the acquired form the hormonal effects of pregnancy and abdominal wall laxity are proposed as determining factors [5-7]. In addition, failure of fusion of the dorsal mesogastrium during foetal development resulting in the characteristic long vascular pedicle has been attributed [8]. However, the precise aetiology of the wandering spleen is not known [2].

## Case presentation

A 27-year-old woman of Arabic ethnicity, presented to the emergency department with a 24-hour history of central abdominal pain which she described as a tight band spanning from her right lumbar region to her left lumbar flank. The pain was of sudden onset, scored 10/10 and exacerbated by movement and eating. There was associated vomiting; clear vomitus and no haematemesis. Her bowels were opening regularly and there was no reported blood in her stools.

Prior to this, she presented to the emergency department four times within the last year, for a milder pain in her left iliac fossa radiating to her back, described as stabbing in nature. She did not have any lower urinary tract or any gynaecological symptoms. During these admissions she was treated for renal colic.

Her past medical history entailed oesophageal gastric reflux with no history of any connective tissue disorders.

On examination, she appeared in discomfort, was apyrexial but had a tachycardia of 117 beats per minute, with otherwise normal cardiorespiratory function.

Her abdomen was soft, with generalised tenderness, but specifically more in her right iliac fossa and left upper quadrant. Rovsing’s sign was negative and bowel sounds were sluggish.

On admission her haemoglobin was 11.7g/dL, white cell count 16.6×10^9^/L, neutrophils 14.8×10^9^/L, her renal, liver function, amylase and lactate were within normal limits.

An abdominal radiograph showed distended small and large bowel loops in the left upper quadrant and paucity of bowel gas in the rest of the image. A chest radiograph was normal.

She was resuscitated with intravenous fluids, given analgesia, antiemetics and started on antibiotics on the basis of an initial diagnosis of appendicitis, because the most tender point in her abdomen was in the right iliac fossa. However, further imaging was requested as the distribution of large bowel gas on the abdominal radiograph seemed abnormal.

An ultrasound (US) of her abdomen showed free fluid throughout her abdomen and pelvis. In the midline of her abdomen was a homogenous mass which resembled the spleen; no blood flow could be elicited on Doppler. The left upper quadrant contained no splenic tissue.

**Figure 1:**
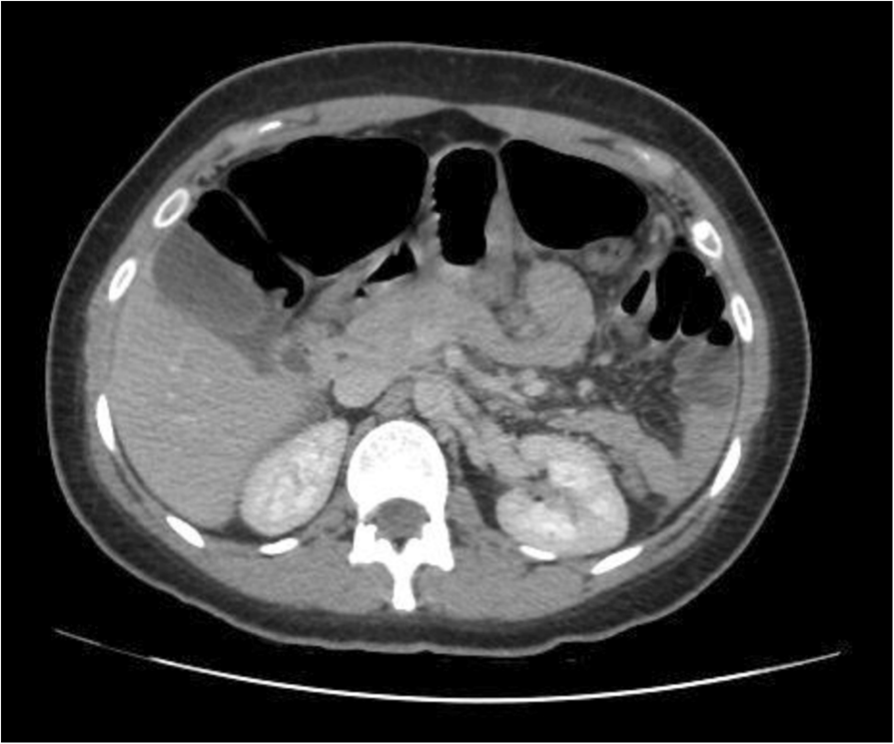
Left upper quadrant filled with small bowel.

**Figure 2:**
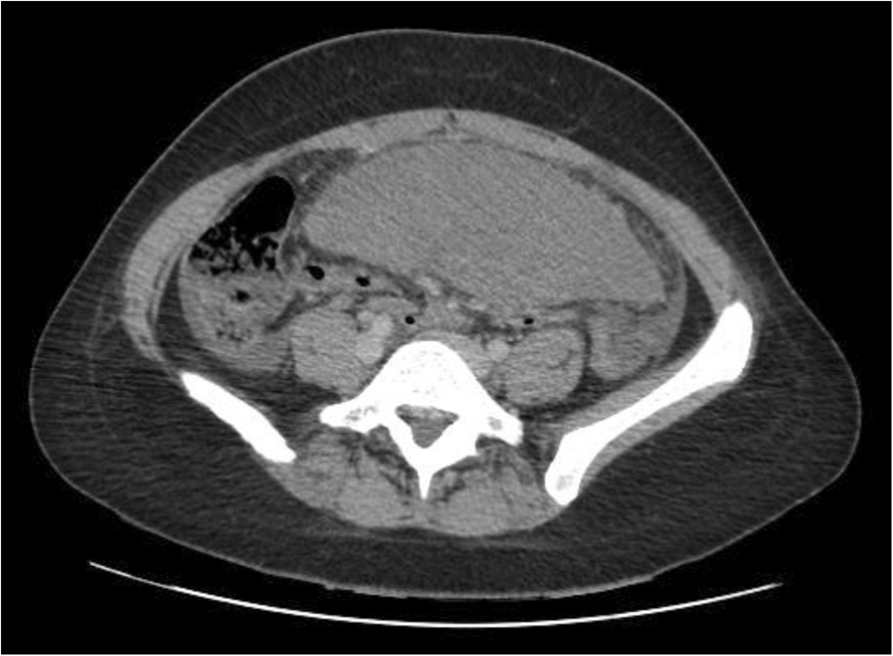
Lower abdomen/pelvis showing spleen.

An urgent computed tomography (CT) was done (Figures 1 and 2) which showed a grossly enlarged spleen at 17cm, with a long mesentery, located in her mid-abdomen. It was torted with resultant splenic infarction. In addition there was swirling of the splenic vessels as they passed inferiorly into her abdomen towards the spleen, which lay above her pelvis. The left upper quadrant showed no splenic tissue but multiple loops of thickened small bowel and free fluid throughout her abdomen and pelvis. Liver, gallbladder, kidneys and adrenals were reported as normal. Her appendix was identifiable and normal, with pelvic organs being unremarkable.

**Figure 3:**
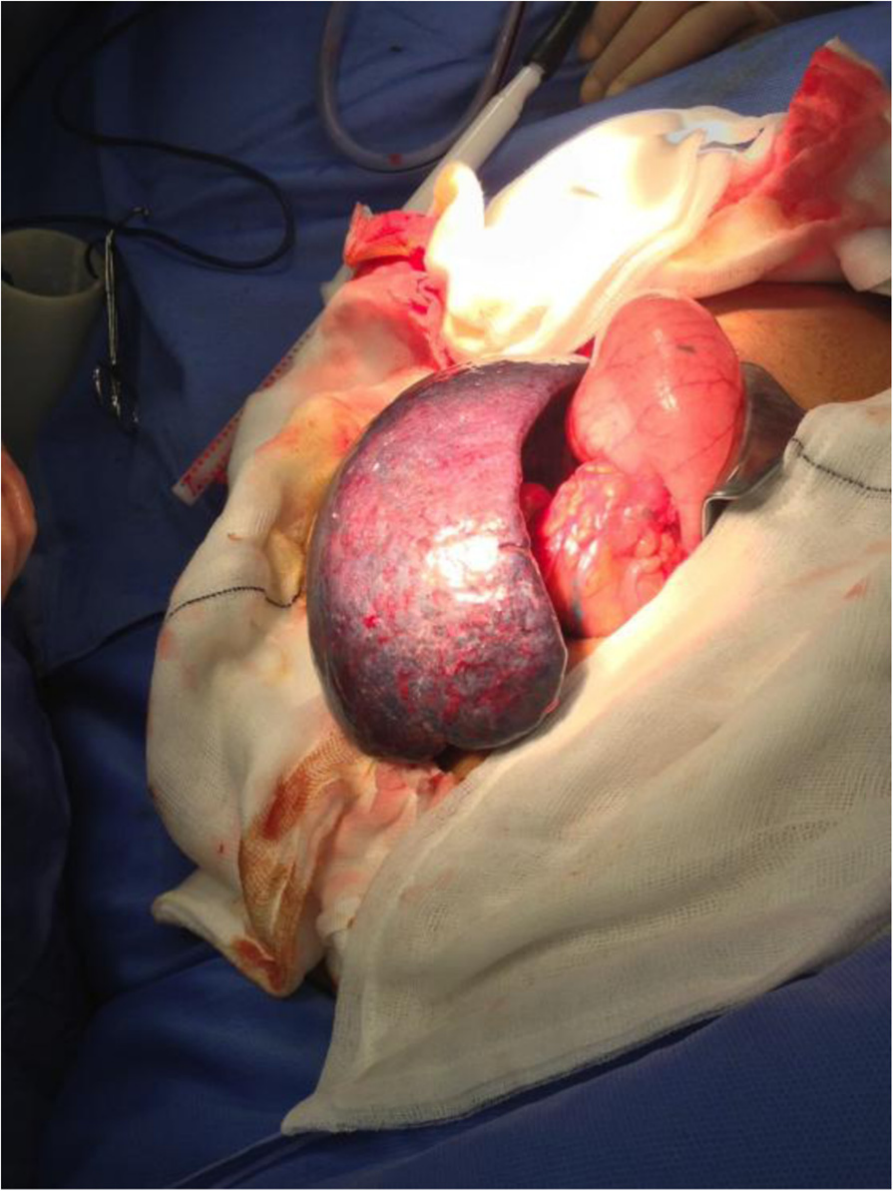
Image of torted spleen.

**Figure 4:**
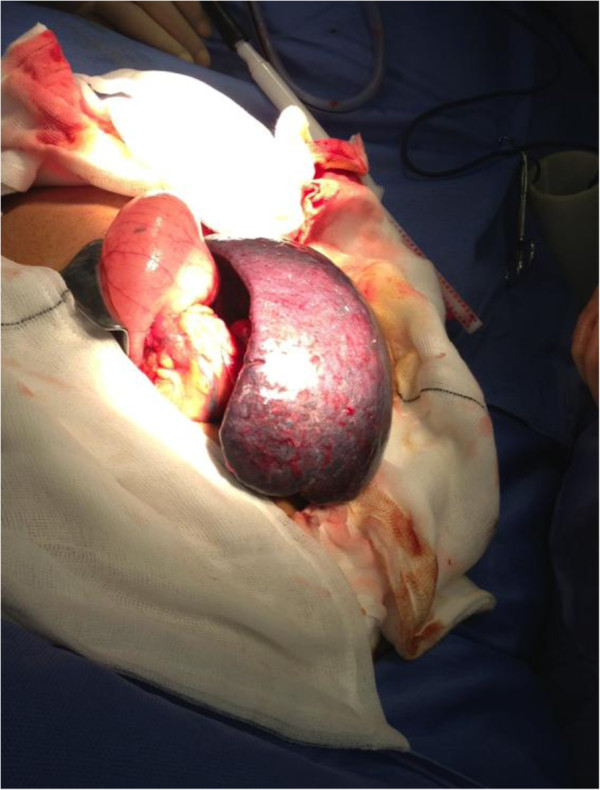
Image of torted spleen.

**Figure 5:**
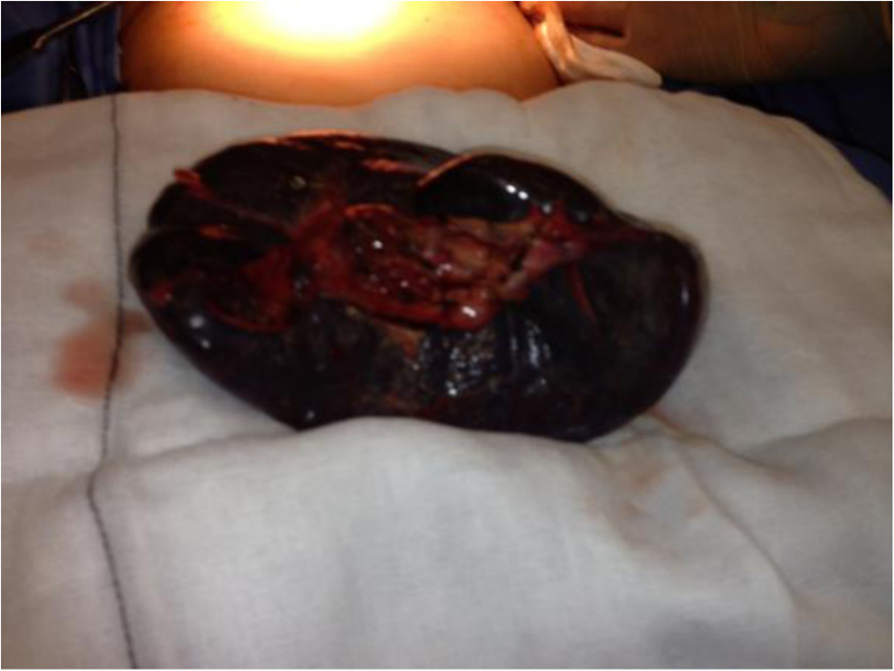
Image of resected spleen.

**Figure 6:**
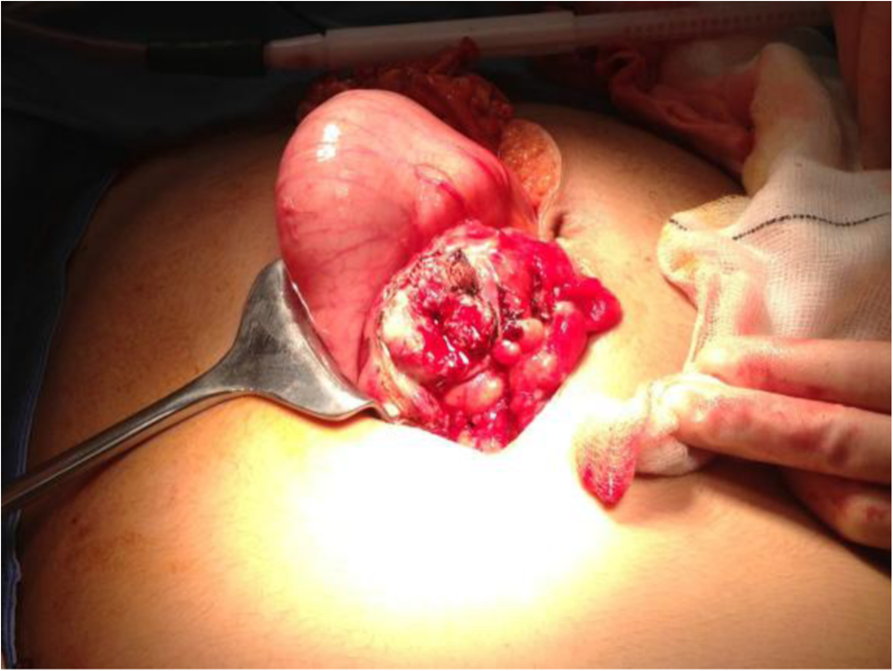
Postsplenectomy.

She was prepared for urgent laparotomy with findings of an infarcted wandering spleen in her mid-abdomen. Her spleen was enlarged to 20cm at its maximum diameter due to venous congestion and resultant infarction. There were no ligamentous attachments to her spleen and the tail of her pancreas was attached to the hilar vessels of her spleen which were on a long mesentery. The infarct was due to 360° twisting of the splenic hilum around the tail of her pancreas. Of note her caecum, right and transverse colon were dilated but there was no obstruction, and her small bowel and appendix were normal (Figures 3, 4, 5 and 6).

Her spleen was unrotated, her hilar vessels were divided close to her spleen with LigaSure™, taking care to preserve the tail of her pancreas, and a splenectomy was performed. The ends of her hilar vessels and pancreatic tail were closed with 1.0 Vicryl sutures. A saline washout was done, and a left Robinson drain was left at the pancreatic tail site, and a right Robinson drain left in her pelvis.

The histology report concluded that within her spleen there was extensive haemorrhagic tissue with a few small residual lymphoid aggregates. A few blood vessels with neutrophilic infiltration were present and the hilum showed blood vessels with minimal intraluminal neutrophilic exudate and neutrophilic infiltration of the vessel wall. In addition there was a surrounding small abscess formation within the hilar tissue. The features were consistent with splenic infarction of ischaemic origin. However, there was no evidence of granuloma or malignancy.

Her postoperative recovery was good; she was started on long-term penicillin V and was given Haemophilius influenza type B, meningococcal and pneumococcus vaccinations 2 weeks after her splenectomy. She was also commenced on long-term aspirin for postsplenectomy thrombocythaemia. The drains were removed after her amylase level was checked and found to be within normal serum levels.

## Discussion

A literature review conducted by Buehner and Baker [3] concluded that patients most commonly presented with: an asymptomatic mass, in the subacute setting with nonspecific gastrointestinal complaints and could also present with an acute abdomen [3]. The use of biochemical blood tests has been found to be nonspecific in terms of helping with diagnosis. [3].

Symptoms may remain quiescent over long periods, but complications are related to torsion or compression of abdominal organs [3]. These can include pancreatitis, bowel obstruction, gastric volvulus, gastric and duodenal compression and most commonly splenic infarction [7]. Splenomegaly is usually a result of torsion of the pedicle and splenic sequestration.

A wandering spleen usually presents between the ages of 20 and 40 years, being more common in women [9,10]. Children make up one-third of cases, with an equal preponderance in boys and girls under 10 years [9].

US imaging with duplex scanning can be used as an initial mode of imaging which can show the position of the wandering spleen with concomitant replacement of bowel in the left upper quadrant [7]. CT contrast imaging is the preferred mode of investigation, with the contrast helping to elucidate the viability of the spleen [6,7]. The most characteristic finding is the absence of the spleen in its normal position and an ectopic mass found somewhere else in the abdomen or pelvis [6]. The whirl sign of the splenic pedicle and surrounding fat is specific for splenic torsion as was the case with our patient [5,6].

Splenopexy is the choice of treatment if the spleen is not infarcted but a splenectomy proceeding detorsion is necessary if there is any sign of infarction [3,11-13]. This should be appropriately followed up by the prophylactic vaccines against postsplenectomy sepsis syndrome. Ideally they should be administered before surgery; however, in emergencies this is not always possible.

## Conclusions

The wandering spleen is a rare differential diagnosis of an acute abdomen but must be considered if a patient presents with abdominal pain associated with a palpable mass and displacement of bowel loops to the left upper quadrant. The best method of confirming the diagnosis seems to be a CT scan, however, US imaging is an equally helpful modality.

## Consent

Written informed consent was obtained from the patient for publication of this case report and accompanying images. A copy of the written consent is available for review by the Editor-in-Chief of this journal.

## Abbreviations

CT: Computed tomography; US: Ultrasound.

## Competing interests

The authors declare that they have no competing interests.

## Authors’ contributions

AS made substantial contributions to: conception, design, acquisition of data, analysis and interpretation of data and was involved in drafting the manuscript. GS was involved in revising the manuscript critically for important intellectual content. Both authors read and approved the final manuscript.
